# Knowledge, attitudes, and perceptions associated with HPV vaccination among female Korean and Chinese university students

**DOI:** 10.1186/s12905-022-01624-1

**Published:** 2022-02-23

**Authors:** Hae Won Kim, Eun Ju Lee, Young Jin Lee, Seo Yun Kim, You Jing Jin, Youngji Kim, Jung Lim Lee

**Affiliations:** 1 College of Nursing, The Research Institute of Nursing Science, Center for Human-Caring Nurse Leaders for the Future by Brain Korea 21 (BK 21) Four Project Seoul National University, Seoul, Republic of Korea; 2 Department of Nursing, Inha University, Inchon, Republic of Korea; 3 Department of Nursing, College of Life and Health Sciences, Hoseo University, Cheonan, Republic of Korea; 4 College of Nursing, The Research Institute of Nursing Science, Seoul National University, Seoul, Republic of Korea; 5 College of Nursing, Seoul National University, Seoul, Republic of Korea; 6 Department of Nursing, College of Nursing and Health, Kongju National University, Gongju, Republic of Korea; 7 Department of Nursing, Daejeon University, Daejeon, Republic of Korea

**Keywords:** Human papillomavirus, Vaccination, Knowledge, Attitude, Perception

## Abstract

**Background:**

Human papillomavirus (HPV) vaccination is a form of primary prevention for cervical cancer. The HPV vaccination rate of female university students is not high in Korea and China. Therefore, the purpose of this study was to identify and compare the factors associated with intention to receive HPV vaccination between Korean and Chinese female university students.

**Methods:**

The participants were 273 Korean and 317 Chinese female university students who had not been vaccinated for HPV, and data were collected using a self-reported questionnaire about attitudes toward HPV vaccination, HPV knowledge, perceptions of HPV infection, and intention to receive HPV vaccine.

**Results:**

There were no significant differences between the Korean and Chinese female university students in HPV knowledge, attitudes, perceptions, and vaccination intention. The factors influencing the intention of HPV vaccination in Korean students were a positive attitude toward the HPV vaccine and a high HPV knowledge score. For Chinese students, sexual experience, awareness of genital warts, a positive attitude toward the HPV vaccine, a high HPV knowledge scores, a perception of the seriousness of HPV infection, and negative emotions regarding HPV infection were significant factors.

**Conclusions:**

It is important to improve attitudes and knowledge about HPV and the HPV vaccine in order to enhance HPV vaccination both in Korea and China. Perceived seriousness and negative emotions regarding HPV infection should be used as a framework to develop subject-tailored interventions in China.

## Background

Human papillomavirus (HPV) is a very common sexually transmitted infection worldwide, and persistent genital infection with high-risk HPV types can lead to the development of anogenital precancers and cancers [1]. Cervical cancer comprised 84% of all HPV-related cancers and ranked in the top three and four cancers affecting women younger than 45 years in China and Korea, respectively [1, 2]. In 2018, China contributed 35% to the global burden of cervical cancer cases and deaths. The age-specific incidence rate of cervical cancer starts rising after 25 years globally [2]. Both in China and Korea, the incidence rate of cervical cancer among young women has increased, resulting in a greater loss of life-years when considering age-weighting [3]. HPV vaccination as a primary preventive intervention plays a key role in decreasing the incidence of cervical cancer. It is necessary to develop effective strategies to improve HPV vaccination rates, particularly in younger age groups.

By 2017, 30 of the 35 Organization for Economic Co-operation and Development countries had introduced HPV vaccination into the national immunization program for girls [4]. In Korea, the first HPV vaccine was approved in 2008 and HPV vaccines were designated as a national immunization program in June 2016 [4]. In China, a bivalent vaccine, a quadrivalent vaccine, and a 9-valent vaccine were approved in 2016, 2017, and 2018, respectively. According to WHO recommendations, the primary target population for HPV vaccination is girls aged 9–14 years, before sexual contact [1]. The Korea Disease Control and Prevention Agency recommend routine immunization for 11–12-year-old girls and catch-up vaccination for females aged 13–26 [4]. In China, each of the three vaccines was licensed for different age ranges from 9 to 45 [5]. In Korea, the prevalence of HPV infection was 34.2% from 2006 to 2011 and the highest HPV infection rate (49.9%) was found among women between the ages of 18 and 29 years [6]. China also showed similar results, with the HPV infection rate peaking between 20 and 24 years at 24.3% [7]. This age group is the secondary target population for HPV vaccination and includes university students. Many university students are living away from parental supervision for the first time and are likely to be exposed to an environment in which they may engage in unprotected high-risk sexual behaviours. College-age women should learn to take the initiative in managing their health. Therefore, health promotion interventions for the prevention of cervical cancer tailored to college students should be established.

The HPV vaccination rate of Korean female university students has been reported to be 12–28% [8–10] and that of Chinese female university students has been reported to be 9.5% [11]. Given the low vaccination rate in both countries, raising the HPV vaccination rate should be an urgent task for women’s health promotion. Behavioural intentions are the immediate antecedent of behaviour, and therefore are a strong predictor of behaviour [12]. Identifying the factors influencing both vaccine uptake and willingness to be vaccinated could be the first step towards developing policies and programs to increase the HPV vaccination rate.

Vaccine hesitancy is strongly associated with the decision of vaccine acceptance, and knowledge, attitudes, and beliefs are factors significantly related to vaccine hesitancy [13, 14]. High knowledge and awareness about HPV infections and HPV vaccination play an important role in HPV vaccine uptake and intention to receive the vaccination [5, 8, 15, 16]. Positive attitudes toward HPV vaccination are a major factor influencing the intention of HPV vaccination [17]. Within the framework of the health belief model, perceived susceptibility, perceived seriousness, perceived benefits, and perceived barriers are significant factors influencing intention to receive the HPV vaccination [5, 9]. Therefore, when investigating intention-related factors, it is important to consider knowledge, attitudes, and perceptions about HPV infection and HPV vaccination in an integrated way.

According to statistics in 2019, the proportion of Chinese students among international students in Korea was 44.4%, accounting for the largest percentage. The proportion of Korean students studying in China among Korean students abroad was 23.8%, and China ranked in the top two international destinations for Korean students [18]. Exchange of college students between both countries is expected to increase further in the future. HPV is transmitted by sexual behaviour and this subject should therefore be approached with due consideration of the socio-cultural context. This study is expected to provide meaningful data suggesting the importance of preventive actions by comparing and analysing knowledge, attitudes, and perceptions of cervical cancer vaccination among Korean and Chinese female college students. Through this research, we intend to provide basic data for establishing culturally sensitive HPV vaccination programs for cervical cancer prevention and women’s health promotion for Korean and Chinese female college students.

## Methods

### Study design, setting, and participants

This study was conducted using a cross-sectional survey. The participants of this study were selected through convenience sampling and recruited at C university in Korea and Y university, a sister university of C university, in China. The inclusion criteria were female university students (1) who were not married and had not been vaccinated against HPV and (2) who were adults (age 19 or older in Korea and age 18 or older in China). The sample size for multiple logistic regression was calculated using the G*Power version 3.1 program. For sample size calculation, the odds ratio (OR) of 1.47–2.23 in previous studies [5, 19] was used as a reference because knowledge is a predictor of HPV vaccination intention. To achieve 5% alpha and 85% power, we used an OR of 1.47, and the required sample size was 262. Considering a 20% withdrawal rate, 327 questionnaires were distributed in Korea and China, respectively. Of those, 273 Korean participants and 317 Chinese participants were included in the final analysis.

### Measurements

The questionnaire consisted of the following topics: general characteristics of subjects, positive attitude toward the HPV vaccine, knowledge about HPV, perceptions of HPV infection, and intention to receive HPV vaccination.

#### General characteristics

Nine questions elicited information on participants’ age, religion, history of smoking and alcohol use, sexual experience, and history of sexually transmitted infections (STIs), as well as whether they had heard of HPV and genital warts before the survey and whether they were possible candidates for HPV vaccination.

#### Positive attitudes toward the HPV vaccine

The instrument for assessing positive attitudes toward the HPV vaccination was developed by the researchers and consists of three items on the beneficial effects of the HPV vaccine (asking about the effect of the HPV vaccine on STI or cervical cancer prevention) and its safety (asking about trustfulness in vaccine safety). The responses to each item are on a 5-point Likert scale and the range of scores is 3–15. Higher scores reflect a more positive attitude toward HPV vaccine. The Cronbach α in this study was 0.80.

#### Knowledge about HPV

HPV knowledge was measured through the tool developed by Kim [19]. The scale consists of eight items that assess knowledge of the cause, symptoms, treatment, and prevention of HPV. Each item is answered “yes”, “no”, or “don’t know”. A correct answer is scored 1 point and a wrong answer or “don’t know” is given 0 point. The range of scores is 0 to 8 and a high score indicates a high knowledge level. The Kuder–Richardson 20 in this study was 0.86.

#### Perceptions of HPV infection

Perceptions of HPV infection were assessed through a tool developed by the researchers based on previous studies [20, 21]. The tool consists of six items with three subscales: two items on seriousness, two items on susceptibility, and two items on negative emotion related to HPV infection. Items are scored using a 5-point Likert scale and the range of each subscale is 2–10. Higher scores indicate more negative perceptions about HPV infection. In this study, the Cronbach α was 0.86 for seriousness, 0.89 for susceptibility, and 0.62 for negative emotions related to HPV infection.

#### Intention of HPV vaccination

The scale used to assess the likelihood of positive HPV vaccination intentions was developed by researchers. The tool consists of six items that directly assess willingness to receive the vaccine (1) in the near future, (2) if it is recommended by one’s parents, a health professional, one’s sexual partner, or one’s friends, and (3) if financial support is available. Each item is answered on a 5-point Likert scale and the range of possible total scores is 6–30. The Cronbach α in this study was 0.91.

### Ethical considerations

This study was approved by the Institutional Review Board (IRB) of C university (no. CKU-15-01-0109) in Korea. For Chinese participants, we received the permission to conduct this survey from J university, which stated that IRB approval was not necessary. Study participants read and signed the informed consent form in Korean or Chinese depending on their nationality. Potential study participants were informed that study participation was voluntary and that they could withdraw their decision to participate at any time. Participants were also informed that the data would be collected anonymously and used only for this study.

### Data collection

The survey was conducted from November 10, 2015, to December 21, 2015. A pilot study was not conducted. The researcher or research assistant visited several departmental offices to explain the purpose of the study and recruited participants by posting a recruitment notice on the office message board. The researcher or research assistant explained the study to each student, giving them information on the study and its purpose. After participants voluntarily signed a consent form, we distributed questionnaires in an envelope individually and collected them in the same way to ensure absolute confidentiality of the research data. We informed the participants that they could skip any item that they could not answer and proceed to the next question. Prior to answering the questionnaire, the following simple information about HPV vaccination was provided: “the HPV vaccine is currently administered via intramuscular injection, and was developed to prevent cervical cancer and a sexually transmitted infection”. It took 15 min for each participant to finish the questionnaire. Participants were provided small stationary as a gift after finishing the questionnaire.

### Data analysis

All statistical analyses were performed using SPSS version 23.0 (IBM Corp., Armonk, NY, USA). The chi-square test, t-test, and Mann–Whitney test were utilized to compare general characteristics; attitudes, knowledge, and perceptions of HPV; and intention of HPV vaccination between Korean and Chinese participants. The influencing factors of the intention of HPV vaccination were determined by logistic multivariate regression, in which adjusted odds ratios (AORs) and their corresponding 95% CIs were calculated. Statistical significance was set at *p* < 0.05 (2-tailed test). The dependent variable (intention) was converted into a dichotomous scale according to the mean scores of total intention, as follows: 0 for scores of 6–21, corresponding to no intention (responses of “absolutely do not intend”, “do not intend”, and “don’t know”), and 1 for scores of 22–30, corresponding to intention(responses of “intend” and “very much intend”) for both Korean and Chinese students.

## Results

### Differences in general characteristics between Korean and Chinese students

Table 1 shows participants’ characteristics and differences by nationality. The response rate of Korean students was 83.5% and that of Chinese students was 96.9%. The participants included 273 Koreans (46.3%) and 317 Chinese (53.7%), with an average age of 20.35 years (range 18–24 years). The mean age was 20.51 ± 0.82 years for the Korean students and 20.22 ± 1.32 years for the Chinese students. Regarding smoking and alcohol drinking, the percentage of Korean students who reported current smoking (13.2%) and drinking (94.1%) was significantly higher than that of Chinese students who reported smoking (5.4%) and drinking (36.6%). The number of Korean students with sexual experience was 30 (11.0%) which was a significantly higher number than reported among the Chinese students (14 persons, 4.4%). The number of Korean students reported STI was 1 (0.4%) and that of Chinese students was 6 (1.9%). However, there was no significant difference in STI experience by nationality.

**Table 1:** General characteristics by nationality

Variable	Category	Total (n = 590)	*n* (%) or mean ± SD		*χ*^2^ (*p*) or *t* (*p*)
			Korean (n = 273)	Chinese (n = 317)	
Age (years)	Min–Max	20.35 ± 1.1	20.51 ± 0.82	20.22 ± 1.32	10.01 (0.002)
		18-24	19–23	18–24	
Religion (n = 589)	No	475 (80.6)	174 (64.0)	301 (95.0)	90.03 (< 0.001)
	Yes	114 (19.4)	98 (36.0)	16 (5.0)	
Smoking (n = 589)	No	536 (91.0)	236 (86.8)	300 (94.6)	11.08 (0.001)
	Yes	53 (9.0)	36 (13.2)	17 (5.4)	
Alcohol drinking	No	217 (36.8)	16 (5.9)	201 (63.4)	208.90 (< 0.001)
	Yes	373 (63.2)	257 (94.1)	116 (36.6)	
Sexual experience (n = 588)	No	544 (92.5)	243 (89.0)	301 (95.6)	9.05 (0.003)
	Yes	44 (7.5)	30 (11.0)	14 (4.4)	
STI experience (n = 589)	No	574 (98.8)	268 (99.6)	306 (98.1)	2.92 (0.130)
	Yes	7 (1.2)	1 (0.4)	6 (1.9)	
Have you had ever heard about HPV? (n = 589)	No	453 (76.9)	203 (74.6)	250 (78.9)	1.48 (0.240)
	Yes	136 (23.1)	69 (25.4)	67 (21.1)	
Have you ever heard about genital warts? (n = 589)	No	484 (82.2)	211 (77.6)	273 (86.1)	7.30 (0.009)
	Yes	105 (17.8)	61 (22.4)	44 (13.9)	
Who would need the HPV vaccine?	Only women	98 (16.7)	47 (17.2)	51 (16.3)	19.28 (< 0.001)
	Both women and men	348 (59.4)	183 (67.0)	165 (52.7)	
	No need without infection symptoms	140 (23.9)	43 (15.8)	97 (31.0)	

*N *= 590, STI, Sexually transmitted infection; HPV, Human papillomavirus

There were 69 (25.4%) female college students in Korea and 67 (21.1%) female college students in China who answered “I have heard of HPV before”. There was no significant difference by nationality with regard to this item. Furthermore, 61 (22.4%) Korean students and 44 (13.9%) Chinese students had ever heard of genital warts, and this was a significant difference (*p *= 0.009). When subjects were asked “who needs HPV vaccination?”, 183 (67.0%) Korean students responded with both males and females, 47 (17.2%) responded with only females, and 43 (15.8%) responded that it was not necessary if there were no symptoms of infection. For Chinese students, 165 (52.7%) responded that both men and women should receive HPV vaccination, 97 (31.0%) responded that it was not necessary if there were no symptoms of infection, and 51 (16.3%) responded that only women should be vaccinated. There was a significant difference between Korean students and Chinese students (*p *< 0.001) (Table 1).

#### Comparison of attitudes toward the HPV vaccine, HPV knowledge, perceptions of HPV, and intention of HPV vaccination between Korean and Chinese students

The results of the analysis of attitudes, knowledge, and perceptions about HPV and the HPV vaccine are shown in Table 2. The average score of Korean students for attitudes toward the HPV vaccine was 10.26 ± 1.87 points (range 3–15 points) and the score of Chinese students was 10.29 ± 1.82 points. The difference between the two groups was not significant. In terms of the subdomains, no significant difference was found regarding the beneficial effects of the HPV vaccine, while recognition of HPV vaccine safety was higher among Chinese students than among Korean students (*p *= 0.004).

**Table 2:** Attitudes, knowledge, and perceptions about HPV and the HPV vaccine by nationality

Variables/items	Total (n = 590)	Korean (n = 273)	Chinese (n = 317)	*Z*(*p*)
		Mean ± SD		
Positive attitudes toward the HPV vaccine (total) (min–max: 3–15)	10.28 ± 1.84	10.26 ± 1.87	10.29 ± 1.82	− 0.08 (0.935)
The vaccine would effectively prevent me from getting STI	3.42 ± 0.71	3.45 ± 0.71	3.39 ± 0.71	− 1.21 (0.225)
The vaccine would effectively prevent me from getting cervical cancer	3.49 ± 0.69	3.53 ± 0.70	3.46 ± 0.68	− 1.47 (0.141)
I trust the vaccine safety	3.37 ± 0.77	3.27 ± 0.74	3.45 ± 0.79	− 2.92 (0.004)
HPV knowledge (total) (min–max: 0–8)	1.90 ± 1.93	1.92 ± 1.98	1.89 ± 1.89	− 0.02 (0.987)
HPV has no symptoms	0.23 ± 0.42	0.22 ± 0.41	0.25 ± 0.43	− 0.95 (0.344)
HPV could disappear without treatment	0.10 ± 0.30	0.12 ± 0.33	0.08 ± 0.27	− 1.83 (0.067)
Vaccination can prevent HPV infection	0.48 ± 0.50	0.45 ± 0.50	0.50 ± 0.50	− 1.19 (0.236)
Using a condom can absolutely prevent HPV infection	0.29 ± 0.46	0.32 ± 0.47	0.27 ± 0.45	− 1.08 (0.281)
HPV only affects women	0.32 ± 0.47	0.34 ± 0.47	0.30 ± 0.46	− 0.89 (0.375)
HPV is affected by coitus	0.30 ± 0.46	0.33 ± 0.47	0.28 ± 0.45	− 1.25 (0.210)
Genital warts induce cervical cancer.	0.05 ± 0.22	0.05 ± 0.21	0.06 ± 0.23	− 0.50 (0.619)
HPV could be completely treated with medication and surgical procedures	0.12 ± 0.32	0.10 ± 0.29	0.14 ± 0.34	− 1.52 (0.128)
Perceptions of HPV				
Subtotal score of seriousness (min–max: 2–10)	7.06 ± 1.43	7.01 ± 1.44	7.11 ± 1.42	− 1.65 (0.100)
Cervical cancer caused by HPV is a serious health problem	3.55 ± 0.77	3.54 ± 0.76	3.56 ± 0.78	− 1.23 (0.221)
Genital warts caused by HPV is a serious health problem	3.51 ± 0.76	3.47 ± 0.75	3.55 ± 0.76	− 1.86 (0.063)
Subtotal score of susceptibility (min–max: 2–10)	5.91 ± 1.70	5.94 ± 1.65	5.89 ± 1.75	− 0.18 (0.856)
I would be susceptible to getting cervical cancer because of HPV ara>	2.99 ± 0.91	3.00 ± 0.86	2.98 ± 0.94	− 0.40 (0.693)
I would be susceptible to getting genital warts because of HPV	2.92 ± 0.89	2.94 ± 0.85	2.91 ± 0.93	0.00 (0.999)
Subtotal score of negative emotions (min–max: 2–10)	6.14 ± 1.64	6.02 ± 1.57	6.24 ± 1.70	− 1.75 (0.081)
I am anxious about getting HPV infection	2.96 ± 0.99	2.88 ± 0.96	3.04 ± 1.01	− 1.98 (0.048)
It would be shameful to get HPV infection	3.17 ± 0.94	3.14 ± 0.89	3.20 ± 0.98	− 1.32 (0.188)

*N*=590. STI, Sexually transmitted infection; HPV, Human papillomavirus

The total possible score for knowledge of HPV ranged from 0 to 7 points, and the mean score of Korean respondents for HPV knowledge was 1.92 ± 1.98 points, which was not significantly different from Chinese respondents’ mean score of 1.89 ± 1.89 points. The total score for each individual item on perceptions of HPV ranged from 2 to 10 points, and the mean score for the domain of seriousness was 7.01± 1.44 points in Korean respondents and 7.11 ± 1.42 points in Chinese respondents, showing no significant difference between the two groups. The mean score for the domain of susceptibility was 5.94± 1.65 points in Korean participants and 5.89± 1.75 points in Chinese participants, and there was no significant difference between the two groups. The mean score for the domain of negative emotions was 6.02 ± 0.89 points in Korean respondents and 6.24 ± 1.70 points in Chinese respondents, again showing no significant difference between the two groups. However, for the item “I am anxious about becoming infected with HPV,” the Chinese respondents showed a significantly higher score (3.04 ± 1.01 points) than their Korean counterparts’ score of 2.88± 0.96 points (*p *= 0.048) (Table 2).

### Comparison of intention of HPV vaccination between Korean and Chinese students

The total scores for HPV vaccination intention ranged from 6 to 30 points, and the mean score was 21.24 ± 4.45 points in Korean respondents and 21.63 ± 4.09 points in Chinese respondents. For the item “In the near future, I intend to receive HPV vaccination”, Chinese students showed a significantly higher intention, with a mean score of’ 3.41 ± 0.81 out of 5 points, compared to the score of 3.33 ± 0.83 points in Korean students (*p *< 0.001). For the item “If my friend receives the vaccination, I intend to receive HPV vaccination”, Chinese students also expressed a significantly higher intention, with a mean score of’ 3.60 ± 0.77 out of 5 points, compared to 3.31 ± 0.87 points in Korean students (*p *< 0.001). However, for the item “If financial support is available, I intend to receive HPV vaccination”, Korean students showed a significantly higher intention, with a mean score of 3.73 ± 0.98 out of 5 points, compared to 3.58 ± 0.85 points in Chinese students (*p *= 0.013) (Table 3).

**Table 3:** Intention of HPV vaccination by nationality

Items	Total (n = 590)	Korean (n = 273)	Chinese (n = 317)	*Z* (*p*) or *t* (*p*)
		*n* (%) or mean ± SD		
I intend to receive HPV vaccination,				
In the near future	3.37 ± 0.82	3.33 ± 0.83	3.41 ± 0.81	− 4.28 (< 0.001)
If my parents recommend it	3.73 ± 0.83	3.74 ± 0.84	3.72 ± 0.83	− 1.30 (0.194)
If a health professional recommends it	3.75 ± 0.87	3.77 ± 0.89	3.73 ± 0.86	− 0.49 (0.625)
If my sexual partner recommends it	3.49 ± 0.87	3.36 ± 0.91	3.61 ± 0.83	− 0.81 (0.419)
If my friends receive the vaccination	3.47 ± 0.83	3.31 ± 0.87	3.60 ± 0.77	− 4.05 (< 0.001)
If financial support is available	3.65 ± 0.91	3.73 ± 0.98	3.58 ± 0.85	− 2.48 (0.013)
Total scores of intention (min–max; 6–30)	21.44 ± 4.27	21.24 ± 4.45	21.63 ± 4.09	− 0.76 (0.446)

*N* = 590, HPV, Human papillomavirus

### Factors Influencing Intention of HPV Vaccination in Korean and Chinese Students

Table 4 summarizes the results of a multiple logistic regression analysis of HPV vaccination intention and presents predictor variables of general characteristics, attitude, knowledge, and subcategories of perceptions. For Korean respondents, a positive attitude toward the HPV vaccine (adjusted OR [AOR] = 1.49, 95% CI 1.22–1.83) and high knowledge score (AOR = 1.20, 95% CI 1.02–1.42) influenced their intention of HPV vaccination.

**Table 4:** Factors influencing intention of HPV vaccination by nationalit*y*

Factor	Intention to receive HPV vaccination	
	Korean students AOR (95%CI)	Chinese students AOR (95% CI)
Age (ref, < 21 years)	1.00 (0.71–1.42)	0.98 (0.78–1.23)
Religion (ref, no)	1.18 (0.66–2.12)	1.78 (0.47–6.71)
Smoking (ref, no)	0.81 (0.35–1.88)	1.19 (0.25–5.72)
Alcohol consumption (ref, no)	2.93 (0.70–12.22)	0.76 (0.42–1.40)
Sexual experience (ref, no)	2.48 (0.99–6.23)	6.95 (1.06–45.59)*
STI experience (ref, no)	125 (0.00–∞)	0.16 (0.01–2.26)
Have you ever heard about HPV (ref, no)	1.26 (0.62–2.58)	1.30 (0.61–2.74)
Have you ever heard about genital warts (ref, no)	1.41 (0.69–2.91)	5.67 (2.12–15.19)**
Positive attitude toward HPV vaccine	1.49 (1.22–1.83)***	1.51 (1.25–1.83)***
HPV knowledge	1.20 (1.02–1.42)*	1.30 (1.09–1.54)*
Seriousness of HPV infection	1.18 (0.94–1.48)	1.30 (1.05–1.62)*
Susceptibility of HPV infection	0.99 (0.80–1.23)	0.84 (0.70–1.01)
Negative emotions about HPV infection	1.16 (0.94–1.44)	1.31 (1.09–1.58)*

*N*=590. HPV, Human papillomavirus; AOR, Adjusted odds ratio; CI, confidence interval; STI, aexually transmitted infection

*p < 0.05, **p < 0.01, ***p < 0.001

For Chinese respondents, the significant factors associated with intention of HPV vaccination were having sexual experience (AOR = 6.95, 95% CI 1.06–45.59), having heard of genital warts (AOR = 5.67, 95% CI 2.12–15.19), a positive attitude toward HPV vaccine (AOR = 1.51, 95% CI 1.25–1.83), a high HPV knowledge score (AOR = 1.30, 95% CI 1.09–1.54), perception of the seriousness of HPV infection (AOR = 1.30, 95% CI 1.05–1.62), and negative emotions about HPV infection (AOR = 1.31, 95% CI 1.09–1.58).

## Discussion

In this study, both Korean and Chinese female college students showed a moderate level of intention to receive the HPV vaccine. The HPV vaccine intentions of Spanish nursing students increased from 4.14 out of 10 before a survey to 6.02 out of 10 post-survey [22], and those of female college students in Lebanon increased from 5.24 out of 10 before a survey to 6.98 out of 10 post-survey [23]. The results of these studies are similar to those of our study. The results of Korean female college students are similar to those of Han et al. [24] (27.47 out of 35 points), but they reflect an improvement compared to the findings of Kim [19] (25% of respondents) and Lee [17] (3.88 out of 7 points). In prior studies of Chinese female college students, the proportion of respondents expressing that they intended to receive HPV vaccination was 44% in the study of Gu et al. [25] and 55.2% in the study of Lin et al. but Chen et al. [26], who conducted a study among women aged 20–35, reported that the percentage of respondents with vaccination intention was as high as 83.7%, showing that the percentage of respondents reporting vaccination intention varied largely among studies. Considering these levels of intention of HPV vaccination, it is necessary to make further efforts to enhance the intention of HPV vaccination among female college students, who are included in the secondary target population for HPV vaccination in both countries, and to ensure that intention to receive the HPV vaccine will lead to actual uptake of the HPV vaccine. In addition, in the case of China, where these vaccines were recently introduced, ongoing research on HPV vaccination needs to be conducted among female college students in various regions to obtain basic data.

Positive attitudes toward the HPV vaccine and HPV knowledge were identified as significant factors influencing intention of HPV vaccination in Korean female college students, while sexual experience, awareness of genital warts, positive attitudes, HPV knowledge, perceptions of the seriousness of HPV infection, and negative emotions toward HPV infection were identified as significant factors influencing intention of vaccination among Chinese female college students. Positive attitudes and HPV knowledge were common factors between both countries. This is consistent with the findings of previous studies that a more positive attitude toward the HPV vaccine and a higher level of HPV knowledge were associated with a higher level of intention of HPV vaccination [5, 15, 25, 26]. In this regard, it should be noted that the level of HPV knowledge among Korean and Chinese female college students was very low in this study, with a mean score of less than 2 out of 8 points and a correct answer rate of less than 25%. This is a very low level, compared to the correct answer rates of 33.5–58.2% reported in previous studies [5, 8, 27].

In addition, only 23.1% of female college students in the two countries responded that they had heard of HPV, and only 17.8% reported that they had heard of genital warts. Since improving knowledge and awareness can induce changes in attitudes and perceptions, above all, it is necessary to actively implement educational programs that provide accurate knowledge in order to increase HPV vaccination rates. HPV vaccination campaign programs should include a variety of content to meet the needs of the target population and should include information appropriate for their knowledge levels, and it would be an effective strategy to deliver such interventions for college students through the internet and mass media considering their patterns of media consumption [5, 10]. Healthcare providers play an important role in changing knowledge and attitudes about HPV vaccination by participating in educational programs, and concrete recommendations from them have a major influence on the decision to get vaccinated [28]. In addition, since young adults, including college students, are the target population, promotional activities in the form of public service announcements at the national level should be conducted in addition to on-campus activities.

In Chinese female college students, perceptions of the seriousness of HPV infection and negative emotions toward HPV infection were found to have a significant effect on vaccination intention. This result is consistent with the results of prior studies that a higher level of perceived severity among health beliefs was correlated with a higher level of HPV vaccination intention [9, 29].

In this study, negative emotions regarding HPV infection were found to influence the intention to receive the HPV vaccine, but previous studies reported that emotions related to HPV infection, such as shame, anxiety, and depression, did not have an effect on intentions to be tested or vaccinated for HPV [19, 22]. Thus, further studies are needed to determine the effects of negative emotions related to HPV infection, as an STI, on the performance of preventive health behaviours. In contrast to the findings of previous studies [5, 9], the results of the present study indicated that perceived susceptibility, which is the perception that “I can get cervical cancer or genital warts due to HPV”, did not have a significant impact on HPV vaccination intention. This result can be attributed to the low levels of HPV knowledge and awareness among the participants of this study. Perception of seriousness and negative emotions should be taken into consideration when designing interventions that improve HPV vaccination intentions.

In Chinese female college students, sexual experience was significantly related to HPV vaccination intention, but prior studies did not show a significant association between these variables [5, 9]. Yet, regarding this difference in study findings, there are some limitations in explaining the statistical significance of the results because the percentage of respondents reporting sexual experience among the total participants was low in both this study and previous studies. However, in both countries, college students are included in the age group with the highest prevalence of HPV infection [6, 7]. In addition, it should be noted that among the participants of this study, only 4.4% of Chinese female college students reported having sexual experience, but 42% of them responded that they had experienced an STI. Therefore, to prevent HPV infection, there is an imperative need to implement an STI prevention program, including HPV vaccination.

This study had some limitations. Firstly, since the present study examined variables at a single point in time, it should be considered that participants’ characteristics and social environment could have affected the results. Secondly, the participants of this study were selected by convenience sampling among students at a single university in Korea and China, respectively, and the reasons for non-participation were not analyzed; therefore, the representativeness of the sample is limited. Additionally, typical forms of survey bias, such as inaccurate recall and social desirability, should be taken into account. Recall bias in this study is expected to have been minimal because the survey did not require participants to recall events from the distant past. However, since the study contained items on sensitive topics, such as smoking, alcohol drinking, sexual experience, and STI experience, social desirability bias may have affected participants’ responses. Nonetheless, the use of anonymous questionnaires is expected to have minimized this issue. Therefore, some caution should be taken in interpreting the study results. In particular, since HPV vaccines have been recently introduced into China and HPV vaccination has not yet been included in China’s national immunization program, basic data should be gathered by conducting research on vaccination in multiple regions. In addition, to effectively prevent cervical cancer in both countries, active research is required to identify the factors influencing the association between HPV vaccination intention and the likelihood of getting vaccinated against HPV.

## Conclusions

The intention of HPV vaccination was moderate among Korean and Chinese female undergraduate students, despite these participants being in the age range with the highest HPV infection rate. The factors influencing intent to receive HPV vaccination in Korean students were a positive attitude toward a vaccine and a high HPV knowledge score. For Chinese students, sexual experience, awareness of genital warts, positive attitude toward the HPV vaccine, HPV knowledge scores, perceptions of the seriousness of HPV infection, and negative emotions regarding HPV infection were significant factors. This study underscores the need to organize an educational program to improve knowledge and awareness about HPV and HPV vaccines. The findings of this study facilitate the understanding of cultural differences and underscore the need to establish culturally sensitive interventions for women’s health promotion for Korean and Chinese young women.

## Data Availability

The raw data are not publicly available because they contain information that could compromise research participant privacy. Individual consent to share individual data was not obtained.

## Abbreviations

HPV: Human papillomavirus
IRB: Institutional review board
STI: Sexually transmitted infection

## References

[1] World Health Organization (WHO) (2017). Human papillomavirus vaccines: WHO position paper, May 2017. Wkly Epidemiol Rec.

[2] Arbyn M, Weiderpass E, Bruni L, de Sanjosé S, Saraiya M, Ferlay J (2020). Estimates of incidence and mortality of cervical cancer in 2018: a worldwide analysis. Lancet Glob Health.

[3] Li X, Zheng R, Li X, Shan H, Wu Q, Wang Y (2017). Trends of incidence rate and age at diagnosis for cervical cancer in China, from 2000 to 2014. Chin J Cancer Res.

[4] Korea Disease Control and Prevention Agency (KDCA). National immunization program for children. https://nip.cdc.go.kr/irgd/index.html.

[5] Lin Y, Lin Z, He F, Hu Z, Zimet GD, Alias H (2019). Factors influencing intention to obtain the HPV vaccine and acceptability of 2-, 4-and 9-valent HPV vaccines: a study of undergraduate female health sciences students in Fujian, China. Vaccine.

[6] Lee EH, Um TH, Chi HS, Hong YJ, Cha YJ (2012). Prevalence and distribution of human papillomavirus infection in Korean women as determined by restriction fragment mass polymorphism assay. J Korean Med Sci.

[7] Zhou HL, Zhang W, Zhang CJ, Wang SM, Duan YC, Wang JX (2018). Prevalence and distribution of human papillomavirus genotypes in Chinese women between 1991 and 2016: a systematic review. J Infection.

[8] Bang KS, Sung S, Koo B, Kim M, Kim Y, Kim J (2011). Female university students’ HPV-related knowledge and influencing factors on HPV vaccination. J Korean Oncol Nurs.

[9] Kim EJ, Kim SH, Chung HI, Kim A (2016). Factors affecting human papillomavirus vaccination among college nursing students. J Korea Acad Ind Coop Soc.

[10] Xu M, Kim HJ, Lee HS, LeeYJ, Han DY, Park SY (2017). Correlation of HPV vaccination and human papillomavirus infection and vaccine knowledge in university female students. J Nus Assoc Ewha Womans Univ.

[11] Liu Y, Di N, Tao X (2020). Knowledge, practice and attitude towards HPV vaccination among college students in Beijing, China. Hum Vaccin Immunother.

[12] Gerend MA, Shepherd JE (2012). Predicting human papillomavirus vaccine uptake in young adult women: comparing the health belief model and theory of planned behavior. Ann Behav Med.

[13] Della Polla G, Pelullo CP, Napolitano F, Angelillo IF (2020). HPV vaccine hesitancy among parents in Italy: a cross-sectional study. Hum Vaccin Immunother.

[14] Dubé È, Farrands A, Lemaitre T, Boulianne N, Sauvageau C, Boucher FD (2019). Overview of knowledge, attitudes, beliefs, vaccine hesitancy and vaccine acceptance among mothers of infants in Quebec, Canada. Hum Vaccin Immunother.

[15] Zhang Y, Wang Y, Liu L, Fan Y, Liu Z, Wang Y (2016). Awareness and knowledge about human papillomavirus vaccination and its acceptance in China: a meta-analysis of 58 observational studies. BMC Public Health.

[16] He J, He L (2018). Knowledge of HPV and acceptability of HPV vaccine among women in western China: a cross-sectional survey. BMC Women’s Health.

[17] Lee KE (2014). Factors associated with intention to receive human papillomavirus vaccine in undergraduate women: An application of the theory of planned behavior. J Korean Acad Fundam Nurs.

[18] Ministry of Education (MOE). Statistics of overseas (foreign) education. https://www.moe.go.kr/boardCnts/list.do?boardID=350&m=0309&s=moe.

[19] Kim HW (2011). Comparison of factors associated with intention to receive human papillomavirus vaccine between male and female undergraduate students. Korean J Women Health Nurs.

[20] Kim HW (2012). Knowledge about human papillomavirus (HPV), and health beliefs and intention to recommend HPV vaccination for girls and boys among Korean health teachers. Vaccine.

[21] Kim HW (2012). Factors Associated with Human Papillomavirus related stigma, shame, and intent of HPV test. J Korean Acad Nurs.

[22] Villanueva S, Mosteiro-Miguéns DG, Domínguez-Martís EM, López-Ares D, Novío S (2019). Knowledge, attitudes, and intentions towards human papillomavirus vaccination among nursing students in Spain. Int J Environ Res Public Health.

[23] Dany M, Chidia A, Nassar AH (2014). Human papillomavirus vaccination: assessing knowledge, attitudes, and intentions of college female students in Lebanon, a developing country. Vaccine.

[24] Han JY, Kim SY, Lee CM, Jeong CR, Kim S, Sung K (2020). Factors affecting sexual knowledge and attitude on intentions to receive human papilloma virus (HPV) vaccination in male and female university students. Global Health Nurs.

[25] Gu C, Niccolai LM, Yang S, Wang X, Tao L (2015). Human papillomavirus vaccine acceptability among female undergraduate students in China: the role of knowledge and psychosocial factors. J Clin Nurs.

[26] Chen L, Song Y, Ruan G, Zhang Q, Lin F, Zhang J (2018). Knowledge and attitudes regarding HPV and vaccination among Chinese women aged 20 to 35 years in Fujian province: a cross-sectional study. Cancer Control.

[27] Park H (2016). Predictors of HPV vaccination status in female nursing university students: HPV related knowledge and perception. J Korean Soc Sch Health.

[28] Santhanes D, Yong CP, Yap YY, Saw PS, Chaiyakunapruk N (2018). Factors influencing intention to obtain the HPV vaccine in South East Asian and Western Pacific regions: a systematic review and meta-analysis. Sci Rep.

[29] Park JS, Lee EJ (2011). Predictors of human papillomavirus vaccination in female university students. Korean J Women Health Nurs.

